# Preparation and Characterization of Poly(ethyl hydrazide)-Grafted Oil Palm Empty Fruit Bunch Fibre for the Removal of Cu(II) Ions from an Aqueous Environment

**DOI:** 10.3390/molecules18078461

**Published:** 2013-07-18

**Authors:** Ili Syazana Johari, Nor Azah Yusof, Md Jelas Haron, Siti Mariam Mohd Nor

**Affiliations:** 1 Department of Chemistry, Faculty of Science, Universiti Putra Malaysia, 43400 Serdang, Selangor, Malaysia; E-Mails: ili@putra.upm.edu.my (I.S.J.); mariam@science.upm.edu.my (S.M.M.N.); 2 Institute of Advanced Technology, Universiti Putra Malaysia, 43400 Serdang, Selangor, Malaysia; 3 Chemistry Unit, Centre of Foundation Studies for Agricultural Science, Universiti Putra Malaysia, 43400 Serdang, Selangor, Malaysia; E-Mail: mdjelas@gmail.com

**Keywords:** copper, poly(ethyl hydrazide), isotherm, kinetic

## Abstract

Poly(ethyl hydrazide)-grafted oil palm empty fruit bunch fibre (peh-g-opefb) was successfully prepared by heating poly(methyl acrylate)-grafted opefb (pma-g-opefb) at 60 °C for 4 h with a solution of hydrazine hydrate (15% v/v) in ethanol. The Fourier transform infrared spectrum of the product shows a secondary amine peak at 3267 cm^−1^, with amide carbonyl peaks at 1729 cm^−1^ and 1643 cm^−1^. The chelating ability of peh-g-opefb was tested with copper ion in aqueous solution. A batch adsorption study revealed that maximum adsorption of copper ion was achieved at pH 5. An isotherm study showed the adsorption follows a Langmuir model, with a maximum adsorption capacity of 43.48 mg g^−1^ at 25 °C. A kinetic study showed that the adsorption of copper ion rapidly reaches equilibrium and follows a pseudo–second-order kinetic model, with a constant rate of 7.02 × 10^−4^ g mg^−1^ min^−1^ at 25 °C. The Gibbs free energy, ∆G⁰, value is negative, indicating a spontaneous sorption process. Entropy, ∆S⁰, gives a positive value, indicating that the system is becoming increasingly disordered after the adsorption of copper ion. A positive enthalpy value, ∆H⁰, shows that the endothermic process takes place during the adsorption and is more favourable at high temperatures.

## 1. Introduction

In recent years, with industrial activity rapidly expanding the discharge of industrial toxic metals into waterways has become a serious problem. Activities such as mining, metal finishing, electroplating, welding, and alloy manufacturing have been proven to be major sources of heavy metal releases into the environment [1]. An increase in copper in our bodies, for example, will irritate the central nervous system and corrode the gastrointestinal system, accompanied by depression, harming the capillary, hepatic, and renal functions and causing other malfunctions [2]. Moreover, copper, mostly discharged in the form of cupric ion [Cu(II)] has been listed as a priority pollutant by the US Environmental Protection Agency (EPA) [3].

Adsorption using chelating fibres is a popular technique nowadays for heavy metal removal [4] due to its simplicity, low cost, and ability to remove elements efficiently, even at trace levels [5]. The application of biosorption to environmental treatment has become a significant research area in the past 10 years. The biosorption of heavy metals from aqueous solutions is a relatively new process that has been proven to be very promising in the removal of contaminants from aqueous effluents. In adsorption, the type of adsorbent is an important factor in effectively removing metal ions. Synthetic resins that can form chelate structures with metal ions have often been used by many researchers for the removal and recovery of heavy metal ions from wastewater. In many cases, petroleum-based synthetic copolymers such as styrene-divinylbenzene have been utilized, but most petroleum-based synthetic polymers resin are in bead form and are neither renewable nor biodegradable. Biomass, which is more biodegradable, could thus be an economical alternative material for heavy metal removal from wastewater. Oil palm empty fruit bunch (opefb), rubber wood, and kenaf are several types of cheap biomass fibre available in Malaysia. Malaysia is one of the World’s major oil palm producers and opefb is one of the major solid wastes left after the oil extraction process; therefore the expected solid waste generated is enough to cause a waste management problem. For this reason, it is worth exploring and modifying agricultural materials for metal adsorption purposes.

In this study, poly(methyl acrylate)-grafted opefb (pma-g-opefb) obtained from the graft copolymerization process was modified to yield poly(ethyl hydrazide)-grafted opefb (peh-g-opefb), which was then used as an adsorbent for the removal of copper from aqueous solutions.

## 2. Results and Discussion

### 2.1. Graft Copolymerization of Methyl Acrylate onto Opefb

**Scheme 1:**
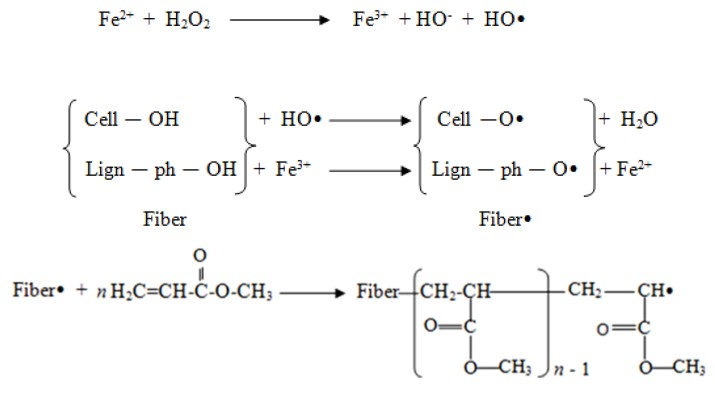
Proposed reaction mechanism in the graft copolymerization of methyl acrylate onto opefb [6].

Hydrogen peroxide was used in a graft copolymerization reaction as an initiator, while ferrous ammonium sulphate was used as a co-initiator. The addition of Fe^2+^ in the reaction produces a hydroxyl radicals during the decomposition of hydrogen peroxide, which leads to the initiation of fibre macroradicals [6]. The macroradicals formed were then reacted with methyl acrylate to form the copolymer poly(methyl acrylate). The process was terminated when two radicals combined and formed a homopolymer and a copolymer. The opefb was successfully grafted with methyl acrylate to obtain pma-g-opefb, with a 117.24% grafting percentage. Scheme 1 illustrates the mechanism of the graft copolymerization process involving methyl acrylate as a monomer and opefb as proposed [6]. The peh-g-opefb was then obtained by reaction of the pma-g-opefb with hydrazine hydrate. The presence of hydrazide functional group in peh-g-opefb provides better chelation involving the carbonyl oxygen and nitrogen atom with copper ions [7] hence has higher adsorption capacity compared to the unmodified opefb.

### 2.2. Characterization of Peh-g-opefb

#### Fourier Transform Infrared Analysis

**Figure 1:**
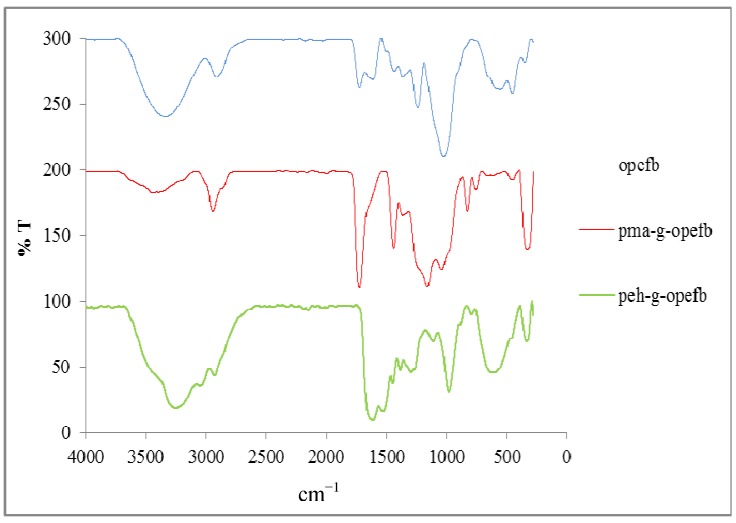
The FTIR spectra for opefb, pma-g-opefb, and peh-g-opefb.

Figure 1 shows the FTIR spectra of all types of opefb, pma-g-opefb, and peh-g-opefb. A broad absorption band appears at around 3,500–3,200 cm^−1^ in the opefb, indicating that the O–H stretching is attributable to the hydroxyl groups of cellulose, absorbed water, hemicelluloses, and lignin [6]. In peh-g-opefb, a broad absorption band appears at about 3300 cm^−1^ and 3100 cm^−1^, indicating N–H stretching from the amine group, consistent with the N–H stretching observed for polyacryl-amidrazone-hydrazide, which appears at 3319 cm^−1^ and 3180 cm^−1^ [3].

The FTIR spectrum of pma-g-opefb shows one absorption band, observed at 1741 cm^−1^, which corresponds to carbonyl stretching of the ester functional groups of the poly(methyl acrylate). When the pma-g-opefb is converted to peh-g-opefb, the carbonyl ester absorption band at 1741 cm^−1^ disappears and a new absorption band appears around 1650 cm^−1^, representing carbonyl stretching from the amide in peh-g-opefb, which may be due to the conversion of the majority of the ester group to hydrazide [8]. Furthermore, another absorption band observed at about 1580 cm^−1^ indicates N–H bending in peh-g-opefb. This result is in agreement with the results of Liu *et al*., who reported the presence of an N–H bending absorption band around 1560 cm^−1^ in poly(acrylaminophosphonic-carboxyl-hydrazide) [4]. The existence of an absorption band around 2900 cm^−1^ in all grafted opefb, ungrafted opefb, and peh-g-opefb may be due to C–H stretching from cellulose and lignin [9].

### 2.3. Copper (II) Ion Adsorption Study

#### 2.3.1. Effect of pH

**Figure 2:**
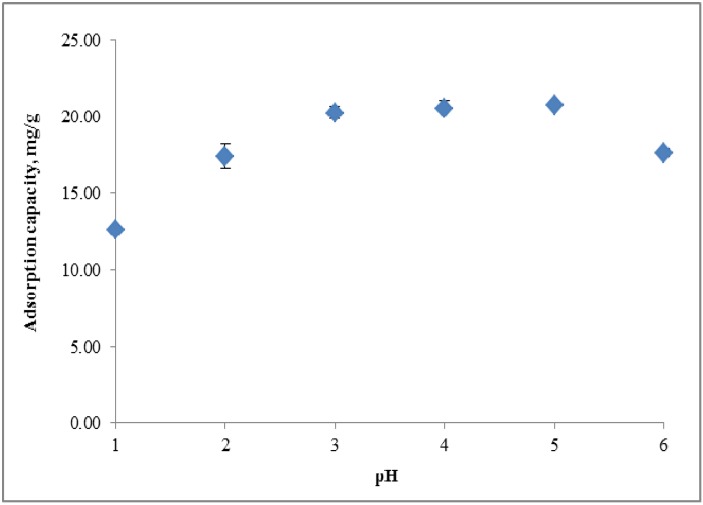
Adsorption capacity of peh-g-opefb towards copper ion at different pH values.

Figure 2 shows that a greater uptake of copper ion was achieved at a pH range of 3–5 with an adsorption capacity of 21.02 mg g^−1^. The graph shows that the adsorption capacity for copper ion increased as the pH increased. The lower uptake of copper ions in acidic media (pH 1 and pH 2) may be attributed to the competition of copper ions and protons with the protonated amine groups, which leads to strong electrostatic repulsion [10,11,12,13]. Hence, the protonated amine group obtained in the lower pH medium reduces the number of hydrazide binding sites available for adsorption with copper ions. As the pH increases, the hydrazides on the adsorbent surface mainly turn into dissociated forms so that the uptake of copper ions increases due to the greater numbers of negatively charged binding sites [13,14,15,16]. As the solution pH increased to pH 6, the adsorption capacity was found to decrease due to the formation of copper hydroxide in solution [17].

#### 2.3.2. Effect of Initial Concentration

**Figure 3:**
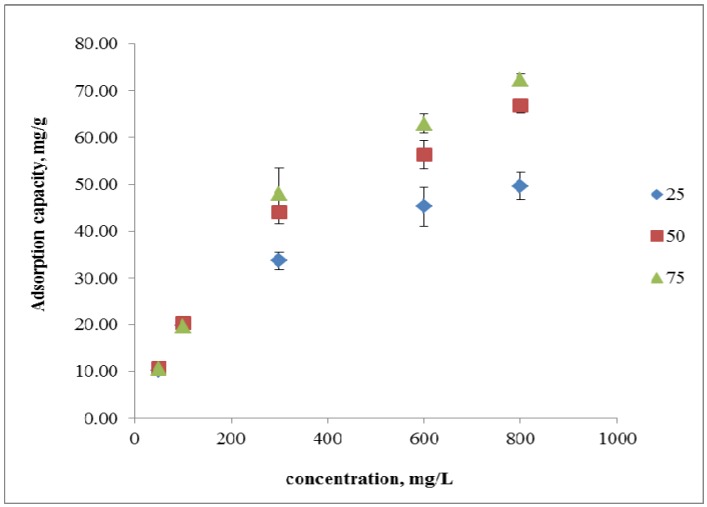
Effect of different initial concentrations at different temperatures on the adsorption capacity of peh-g-opefb for copper ion.

Figure 3 show that the adsorption capacity for copper ions increases from 9.95 mg g^−1^ to 52.98 mg g^−1^ at 25 °C, from 10.23 mg g^−1^ to 65.41 mg g^−1^ at 50 °C, and from 10.64 mg g^−1^ to 72.89 mg g^−1^ at 75 °C as the initial concentration increases from 50 mg L^−1^ to 800 mg L^−1^. However, the percentage removal of copper ions by peh-g-opefb reveals a reversed trend. For a fixed dosage of peh-g-opefb and a fixed-pH copper ion solution, the percentage removal of copper decreases from 97.38% to 14.4% at 25 °C, from 99.29% to 25.21% at 50 °C, and from 96.97% to 34.68% at 75 °C as the initial concentration of metal ions increases.

The initial concentrations of metal ions act as an important driving force to be overcome the total mass transfer resistance of metal ions between the aqueous and solid phases [16,18,19]. The adsorption capacity increases with higher concentrations, suggesting that the increased amount of metal ions in the solution has enhanced the interactions between metal ions and the active sites on the adsorbent surface [20,21]. However, as the concentration of copper increases, the percentage removal decreases, indicating that peh-g-opefb tends to become saturated, yielding no further sites available for adsorption [2,22].

#### 2.3.3. Adsorption Isotherms

Isotherm studies are fundamental in determining the nature of adsorption between an adsorbent and metal ions. They indicate how the metal ions are distributed between adsorbents and metal solutions in the equilibrium phase [2]. To illustrate the data obtained, Langmuir and Freundlich isotherm models were proposed for the adsorption of solute on a solid surface.

The Langmuir isotherm model assumes a homogeneous surface and a constant sorption potential and can be expressed with the following equation:

C_e_/q_e_ = C_e_/Q_o_ + 1/Q_o_b (1)

where Q_o_ (mg g^−1^) is the maximum adsorption at the monolayer, C_e_ (mg L^−1^) is the equilibrium concentration, q_e_ (mg g^−1^) is the amount of copper adsorbed at equilibrium concentration, and b (L g^−1^) is the Langmuir constant.

The Freundlich isotherm model deals with a heterogeneous surface, with assumption that different sites with several adsorption energies are involved, and can be expressed as follows:

Log q_e_ = log K_F_ + log C_e_/n (2)

where K_F_ is the Freundlich constant (mg g^−1^) that indicates sorption capacity and n is the Freundlich constant that indicates adsorption intensity.

Table 1 shows the Langmuir and Freundlich isotherm data of adsorption of copper ion onto peh-g-opefb. From the R^2^ values, it can be concluded that the equilibrium data of copper adsorption fit the Langmuir isotherm model better than the Freundlich model, with correlation coefficients of 0.9645, 0.9863, and 0.9849 at 25, 50, and 75 °C, respectively. Therefore, it can be concluded that copper adsorption onto peh-g-opefb involves a monolayer adsorption with equivalent active sites and a uniform adsorbent surface. Hence, the adsorbed copper or nickel ions do not compete with each other and equilibrium is established where all the adsorbed ions are in contact with the adsorbent’s surface [23].

**Table 1:** Langmuir and Freundlich isotherm constants, maximum adsorption capacities, Q_max_, correlation coefficients, and R^2^ values for copper adsorption at various temperatures.

Langmuir isotherm					Freundlich isotherm		
Temp. (°C)	Q_max_ (mg g^−1^)	b (L mg^−1^)	R^2^	R_L_	K_F_ (mg g^−1^)	n	R^2^
25	43.48	0.3382	0.9645	0.0161	10.87	4.4603	0.9350
50	59.17	0.0817	0.9863	0.0601	13.16	3.7078	0.9990
75	76.92	0.0289	0.9849	0.1460	14.02	4.3687	0.9648

The maximum adsorption capacities of peh-g-opefb with other natural adsorbents are listed in Table 2. Among the studies listed in Table 2, Haron *et al*. [9] also used modified opefb as an adsorbent. The adsorption capacity for copper ion of poly(hydroxamic acid)-grafted opefb is higher than that of peh-grafted opefb obtained in this study. The differences in uptake capacities may be due to the properties of biosorbent materials, such as structure, functional group and surface area, and solution chemistry [24].

**Table 2:** Maximum adsorption capacities (Q_max_) for copper of other low-cost biosorbents.

Adsorbent	q_max_ (mg g^−1^)	References
	Cu(II)	
Opefb (grafted with methyl acrylate and heated with hydrazine hydrate)	43.48	present study
Rape straw	7.72	[19]
*Uncaria gambir* (polymerized by formaldehyde)	9.95	[23]
Cashew nut shell	20.00	[17]
Opefb (grafted with methyl acrylate and reacted with hydroxylammonium chloride)	74.10	[9]
Rubber leaf powder	14.97	[12]
Lentil shell Wheat shell Rice shell	9.59 17.42 2.95	[16]
opefb	3.6	[25]
Palm kernel fibre (treated with HCl)	13.06	[26]

The essential features of the Langmuir isotherm can be expressed in terms of a dimensionless constant separation factor, R_L_, used to predict whether the sorption system is favourable or unfavourable in a batch adsorption process. Using the b value obtained from the Langmuir isotherm equation, one can calculate the separation factor, R_L_, using the following equation:

R_L_ = [1/1 + b C_0_] (3)

where C_0_ is the initial concentration of copper ion (mg L^−1^) and b is the Langmuir constant (L mg^−1^).

The data show that the R_L_ values for copper adsorption onto peh-g-opefb are favourable at all temperatures, for all R_L_ values between zero and one. In addition, the *n* value obtained from the Freundlich equation can also indicate the favourability of the adsorption process: values of *n* from two to 10 are good, values of one to two denote moderate difficulty, and values less than one are poor [27]. Based on the data the values of *n* for copper adsorption onto peh-g-opefb at all temperatures studied were in the range of two to 10, indicating a favourable adsorption process and thus in consistent with the R_L_ values obtained from the Langmuir equation.

#### 2.3.4. Adsorption Thermodynamics

Thermodynamic parameters such as changes in enthalpy (∆H⁰), in entropy (∆S⁰), and in free energy (∆G⁰) are important to determining the spontaneity of the adsorption process. Entropy, ∆S⁰, and enthalpy, ∆H⁰, values are obtained from the slopes and intercepts of the linear regression of the plot ln K_D_ versus 1/T, whereas the free energy is determined using the following equation:

∆G⁰ = ∆H⁰ − T∆S⁰
(4)

where ∆G⁰ is free energy (kJ mol^−1^), ∆H⁰ is standard enthalpy (kJ mol^−1^), ∆S⁰ is standard entropy (J mol^−1^ K^−1^), and T is temperature (K).

The summarized values of all the thermodynamic parameters for copper adsorption are tabulated in Table 3.

**Table 3:** Thermodynamic parameters for copper adsorption by peh-g-opefb.

Temp, K	E nthalpy, ∆H⁰ (kJ mol^−1^)	Entropy, ∆S⁰ (J mol^−1^ K^−1^)	Free energy, −∆G⁰ (kJ mol^−1^)
298	20.96	87.75	5.19
323			7.38
348			9.57

The negative values of free energy, ∆G⁰, indicate the spontaneous nature of the adsorption process. The ∆G⁰ values for copper adsorption increase as the temperature increases, which leads to higher adsorption capacities [28] and increases the feasibility of adsorption at higher temperatures [29]. In other words, more negative values reflect a more energetically favourable adsorption process [30]. The positive value of entropy, ∆S⁰, indicates that the system is becoming more disordered and increasingly random at the solid–solution interface during adsorption [31]. The positive value of enthalpy, ∆H⁰, shows that the adsorption of copper ions onto peh-g-opefb involves an endothermic adsorption process.

#### 2.3.5. Adsorption Kinetics

**Figure 4:**
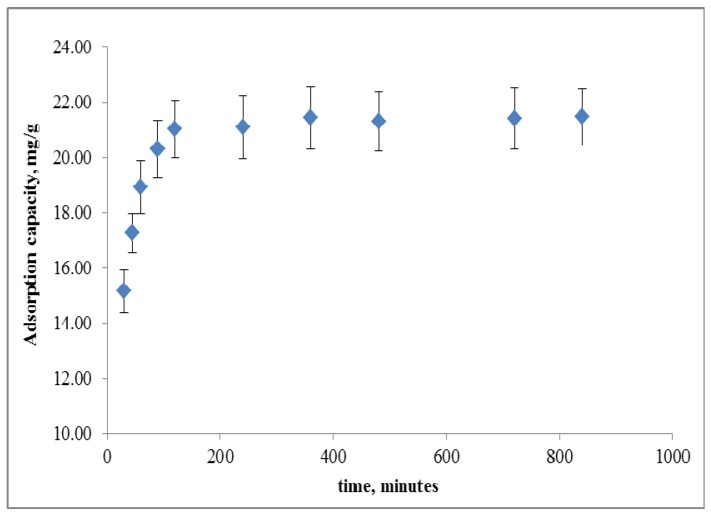
Adsorption capacity for copper ion by peh-grafted opefb for different contact times.

Figure 4 shows that the adsorption of copper rapidly increases with increasing contact time from zero to 120 min and then gradually increases before reaching an equilibrium stage at approximately 480 min.

To determine the mechanism and constant characteristics of sorption, two kinetic models were applied to the experimental data: a pseudo–first-order kinetic model and a pseudo–second-order kinetic model. The pseudo–first-order kinetic model is expressed as the following equation:

ln(q_e_−q_t_) = ln q_e_ − k_1_t/2.303 (5)

where q_t_ is the amount of adsorbate (mg g^−1^) on the sorbent at time t, q_e_ is the amount of adsorbate (mg g^−1^) on the sorbent at equilibrium, and k_1_ is the first-order rate constant (min^−1^).

Ho and McKay [32] proposed the pseudo–second-order kinetic model based on the assumption that the adsorption process follows second-order chemisorption. The pseudo–second-order kinetic model can be written as the following equation:

t/q_t_ = 1/k_2_q_e_^2^ + t/q_e_ (6)

where q_t_ is the amount of adsorbate (mg g^−1^) on the sorbent at time t, q_e_ is the amount of adsorbate (mg g^−1^) on the sorbent at equilibrium, and k_2_ is the second-order rate constant [g(mg min)^−1^].

The data show that the adsorption capacities, q_e_, are much lower than the experimental q_e_ for the pseudo–first-order model for copper adsorption, indicating that copper adsorption does not occur exclusively with one site per ion [18,28]. On the other hand, pseudo–second-order kinetic models yield q_e_ values close to the experimental q_e_ for copper adsorption onto peh-g-opefb, with R^2^ values of 0.99, confirming the applicability of a pseudo–second-order kinetic model for adsorption by peh-g-opefb.

It is also can be concluded that the overall rate of copper adsorption is controlled by chemical processes [18]. This result is in agreement with the isotherm data reported earlier. Table 4 summarizes the kinetic data for copper adsorption onto peh-g-opefb.

**Table 4:** Comparison of pseudo–first-order and pseudo–second-order rate constants k and calculated (q_e_^a^) and experimental (q_e_^b^) adsorption capacity values.

Kinetic models	q_e_ exp (mg g^−1^)	Rate constant, k_1_ (min^−1^), k_2_ (g mg^−1^ min^−1^) (×10^−3^)	q_e_ calc. (mg g^−1^)	Correlation coefficient, R^2^
Pseudo-first order	20.33	3.90	4.195	0.8952
Pseudo-second order		2.93	20.79	0.9999

## 3. Experimental

### 3.1. Instruments and Apparatus

An inductively coupled plasma-optical emission spectrometer (ICP-OES; Perkin Elmer Instruments, Waltham, MA, USA) and an FTIR spectrophotometer (Perkin Elmer) were used.

### 3.2. Materials and Reagents

The Wood Chemistry Division of the Forest Research Institute Malaysia (FRIM) provided the opefb fibre. The opefb was soaked with distilled water for 24 h and washed with hot water and acetone to remove impurities. Then the fibre was dried in an oven at 60 °C. Methyl acrylate was purchased from Acros Organics (Waltham, MA, USA). Activated alumina used for the methyl acrylate purification was purchased from Merck (Darmstadt, Germany). Hydrogen peroxide, 30% from Riedel-de-Hazen (Seelze, Germany); ammonium ferrous sulphate from Fluka (Switzerland); Hydrazine hydrate, 100% from Merck (Hohenbrunn, Germany); Absolute ethanol (Merck), and Copper (II) sulphate (HmbG Chemicals) were used as received.

### 3.3. Graft Copolymerization of Pma-g-Opefb Chelating Fibres

The opefb fibre was ground with a stainless steel grinder, washed with hot water, rinsed with acetone, and dried in an oven at 60 °C. The fibre was then sieved using a 180-micron sieve to obtain a homogeneous fibre size. About 30.0 g of opefb was suspended under a nitrogen atmosphere in 500 mL of distilled water containing 20 mL of hydrogen peroxide (6%) as an initiator, 1.60 g of ferrous ammonium sulphate as a co-initiator, and 50 mL of purified methyl acrylate monomer. The mixture was heated to 75 °C for 3 h. After the grafted product was washed with acetone, the pma-g-opefb was dried in an oven. The details of the preparation of pma-g-opefb have been previously described [9]. The final weight was measured and the grafting percentage (P_g_) was calculated using the following formula:

Grafting percentage (P_g_) = (W_2_ − W_1_)/W_1_ × 100 (7)

where W_2_ and W_1_ are the weights of the purified grafted product and the initial weight of the opefb, respectively.

### 3.4. Modification of the Pma-g-Opefb

A total of 1.0 g of pma-g-opefb was heated at 60 °C for 4 h with a hydrazine hydrate solution in ethanol (15% v/v). Then, the final product was filtered and washed several times with ethanol and dried in an oven at 60 °C.

### 3.5. Metal Ion Uptake Experiments Using a Batch Method

#### 3.5.1. Effect of pH

The uptake of metal ions was carried out by placing 0.1 g of peh-g-opefb in a centrifuge tube of 20 mL of metal solution (100 mg L^−1^) at a controlled pH. Sodium acetate was used as a buffer and the pH was adjusted within the range from pH 1 to pH 6, using various concentration of HCl. After adsorption, the sample was filtered and the amount of remaining metal ions in the sample was determined.

#### 3.5.2. Effect of Initial Concentration and Temperature

The effect of the initial concentration of metal ions in solution was evaluated by placing 0.1 g of peh-g-opefb fibre into 20 mL of metal ion solution at different concentrations (50–1,000 mg L^−1^) at a particular pH. The experiment was carried out at different temperatures to determine thermodynamic parameters such as free energy (∆G⁰), entropy (∆S⁰), and enthalpy (∆H⁰).

#### 3.5.3. Kinetic Study

Metal ion uptake was evaluated by placing 0.1 g of peh-g-opefb fibre into 20 mL of metal ion solution (100 mg L^−1^) at different time intervals. After each period, the samples were filtered and the residual solutions were determined using an ICP-OES.

## 4. Conclusions

Biomass, which is more biodegradable, can be an economical alternative material for the removal of heavy metals from wastewater. In this study, opefb fibre was chemically modified to increase the capability of the biomass to adsorb heavy metal ions. Chemically modified opefb (peh-g-opefb) was successfully prepared and applied in the removal of copper ion from aqueous solutions. Maximum copper adsorption onto peh-g-opefb was achieved at around pH 5. The adsorption also fit the Langmuir isotherm model and the second-order kinetic model, indicating a monolayer chemisorption process. Among natural adsorbents, particularly opefb-based adsorbents, peh-g-opefb is one a potential bioadsorbent to be applied in the removal of copper ion in water and wastewater treatment.
